# Crystal structures of 2,3,8,9,14,15-hexa­methyl-5,6,11,12,17,18-hexa­aza­tri­naphthyl­ene and 2,3,8,9,14,15-hexa­phenyl-5,6,11,12,17,18-hexa­za­tri­naphthyl­ene di­chloro­methane disolvate

**DOI:** 10.1107/S2056989018000725

**Published:** 2018-01-16

**Authors:** Pia Fangmann, Marc Schmidtmann, Rüdiger Beckhaus

**Affiliations:** a Institut für Chemie, Fakultät für Mathematik und Naturwissenschaften, Carl von Ossietzky Universit t Oldenburg, 26129 Oldenburg, Germany

**Keywords:** crystal structure, *N*-heterocycles, multidentate ligand, π–π stacking

## Abstract

The crystal structures of two substituted HATN (hexa­aza­tri­naphthyl­ene) derivatives, namely 2,3,8,9,14,15-hexa­methyl- and 2,3,8,9,14,15-hexa­phenyl-5,6,11,12,17,18- hexa­zatri­naphthyl­ene (HATNMe_6_ and HATNPh_6_), are reported. Whereas the structure of the methyl-substituted derivative (HATNMe_6_) contains no solvent mol­ecules (C_30_H_24_N_6_), the hexa­phenyl-substituted structure (HATNPh_6_) contains two mol­ecules of di­chloro­methane (C_60_H_36_N_6_·2CH_2_Cl_2_). This class of planar bridging ligands is known for its electron-deficient systems and its ability to form π–π stacking inter­actions. Indeed, in both crystal structures strong π–π stacking inter­actions are observed, but with different packing features. The di­chloro­methane mol­ecules in the crystal structure of HATNPh_6_ are situated in the voids and are involved in C—H⋯N contacts to the nitro­gen atoms of the pyrazine units.

## Abstract 2

2,3,8,9,14,15-Hexamethyl- and 2,3,8,9,14,15-hexa­phenyl-5,6,11,12,17,18-hexa­zatri­naphthyl­ene (HATNMe_6_ and HATNPh_6_) are derivatives of hexa­aza­tri­naphthyl­ene (HATN). In the crystal structures of the two compounds, pronounced π–π stacking dominates the packing.

## Chemical context   

Over the last decades, hexa­aza­tri­phenyl­ene (HAT) and its derivatives have shown numerous applications in magnetic materials, semiconductors, sensors and polymers for energy storage (Segura *et al.*, 2015 ▸). These electron-deficient, aromatic and planar systems are known for their excellent π–π stacking ability (Alfonso & Stoeckli-Evans, 2001 ▸) and their three potential chelating positions to form metal complexes. Therefore, a variety of metal HAT or HATN (hexa­aza­tri­naphthyl­ene) complexes are known (Kitagawa & Masaoka, 2003 ▸). Complexes with ruthenium (HATN; Ghumaan *et al.*, 2007 ▸), rhenium (HATN; Roy & Kubiak, 2010 ▸), cobalt (HATN; Moilanen *et al.*, 2016 ▸) and titanium (HATNMe_6_; Piglosiewicz *et al.*, 2005 ▸) have been investigated, in particular due to their inter­esting electrochemical, photophysical and magnetic properties. The synthesis, electrochemical and photophysical properties of the title compounds HATNMe_6_ (**1**) (Catalano *et al.*, 1994 ▸; Fraser *et al.*, 2011 ▸) and HATNPh_6_ (**2**) (Gao *et al.*, 2009 ▸) have already been published. Herein we report on the corresponding crystal structures of the two HATN derivatives.

## Structural commentary   

**Figure 1:**
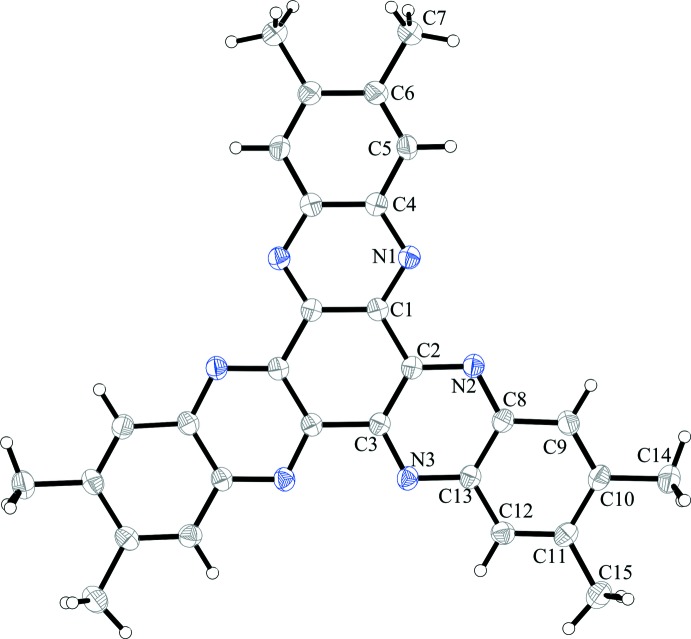
The mol­ecular structure of **1** with the atom labelling and displacement ellipsoids drawn at the 50% probability level. H atoms are given as spheres of arbitrary size. Unlabelled atoms are generated by the symmetry operation (1 − *x*, *y*, 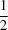 − *z*).

The title compound HATNMe_6_ (**1**) crystallizes without solvent mol­ecules in the ortho­rhom­bic space group *Pbcn* with four formula units per unit cell and half a mol­ecule of HATNMe_6_ in the asymmetric unit, the other half being completed by twofold rotation symmetry (Fig. 1 ▸). The mol­ecule is nearly planar with a slight deviation of the outer annulated benzene rings [2.25 (6)° for C8–C13 and 4.09 (6)° for C4–C6^i^; symmetry code: (i) 1 – *x*, *y*, 1/2 – *z*]. The central six-membered ring of **1** exhibits three longer (C1—C2, C3—C3^i^: average 1.474 Å) and three shorter (C2—C3, C1—C1^i^: average 1.427 Å) C—C bonds. The C—C bonds at the annulated benzene rings show differences in bond lengths. While the outermost bonds (C10—C11 and C6—C6^i^, respectively) are elongated (average 1.438 Å) the bonds to the left and right of these bonds (C5—C6, C9—C10, C11—C12) are shortened (average 1.366 Å).
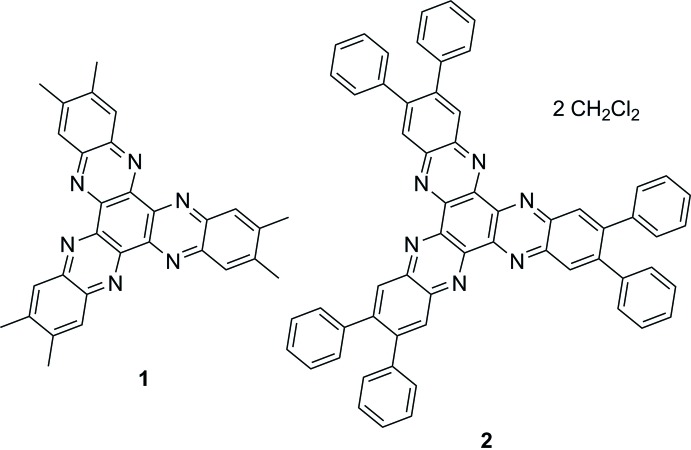


**Figure 2:**
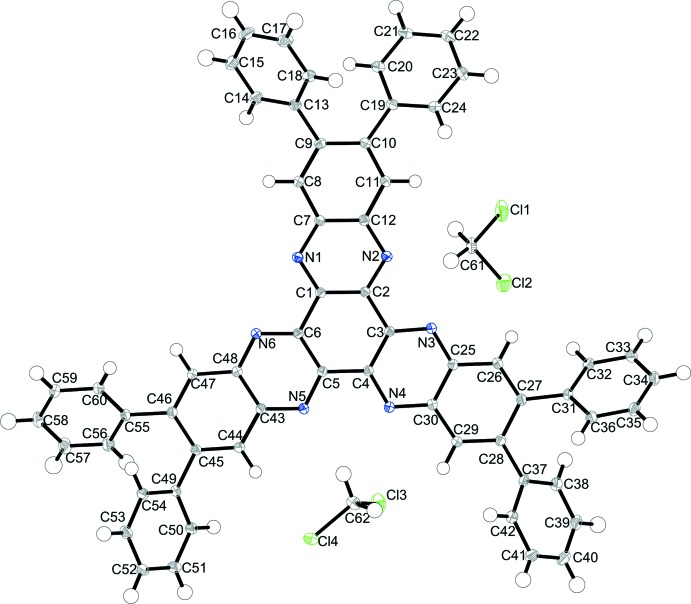
The structures of the mol­ecular entities in **2**. Displacement ellipsoids are drawn at the 50% probability level. H atoms are drawn as spheres of arbitrary size.

HATNPh_6_ (**2**) crystallizes with two mol­ecules of CH_2_Cl_2_ in the triclinic space group *P* 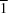 with two formula units per unit cell (Fig. 2 ▸). The mol­ecule is, aside from the terminal phenyl groups, nearly planar with a slight deviation of the outer annulated benzene rings [9.97 (6)° for C43–C48, 8.96 (6)° for C7–C12, and 4.11 (6)° for C25–C30]. The terminal phenyl groups do not lie in this plane and are twisted [dihedral angles between the least-squares planes of the six-membered central ring system and the phenyl rings: 47.60 (7)° for C49–C54, 54.11 (7)° for C55–C60, 32.99 (6)° for C19–C24, 47.26 (6)° for C13–C18, 46.74 (6)° for C31–C36 and 44.26 (7)° for C37–C42]. The central six-membered ring of **2**, like in HATNMe_6_ (**1**), exhibits three longer (C2—C3, C4—C5, C6—C1; average 1.474 Å) and three shorter (C1—C2, C3—C4, C5—C6; average 1.430 Å) C—C bonds. These distances are slightly shorter in comparison with HATN (Alfonso & Stoeckli-Evans, 2001 ▸; average 1.48 and 1.43 Å) but still longer than known for HAT(CONH_2_)_6_ (Beeson *et al.*, 1996 ▸; average 1.46 and 1.41 Å). As has been noted for HATNMe_6_ (**1**) above as well as for HATN (Alfonso & Stoeckli-Evans, 2001 ▸), the annulated benzene ring shows differences in C—C bond lengths. For **2**, the outermost bonds (C9—C10, C27—C28 and C45—C46, respectively) are elongated (average 1.449 Å) and the bonds to the left and right of these bonds (C8—C9, C10—C11, C26—C27, C28—C29, C44—C45, C46—C47) are shortened (average 1.379 Å).

## Supra­molecular features   

**Figure 3:**
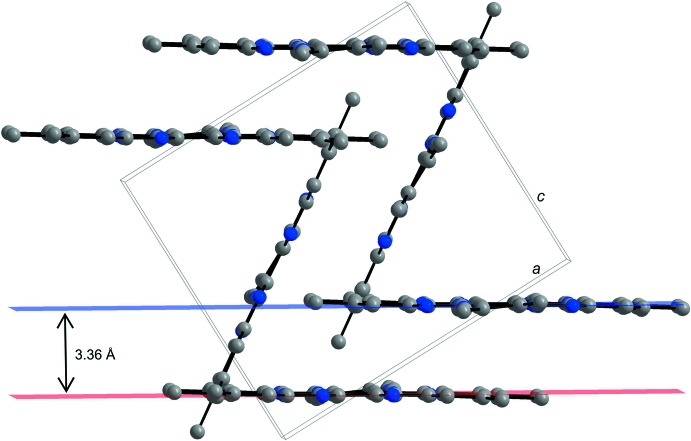
A view along the *b* axis showing parts of the π–π inter­actions between the parallel displaced HATNMe_6_ (**1**) mol­ecules. H atoms have been omitted for clarity. Colour code: C grey, N blue spheres.

As a result of the π–π stacking ability of tri­naphthyl­ene derivatives HATNMe_6_ (**1**) and HATNPh_6_ (**2**), these mol­ecules stack in layers in their respective crystal structures. In the crystal packing of HATNMe_6_ (**1**), a herringbone-like arrangement of mol­ecules is observed (Figs. 3 ▸ and 4 ▸). Individual mol­ecules are arranged in layers and have a short plane–to–plane distance (defined by the central rings) of 3.3602 (5) Å. However, the π–π overlap occurs only in small areas, as shown by the rather large parallel displacement of the mol­ecules with an angle of 31.52° and a shift of 5.48 Å between the centroids. The resulting layers within the herringbone-like structure stack at an angle of 63.1° to each other.

**Figure 5:**
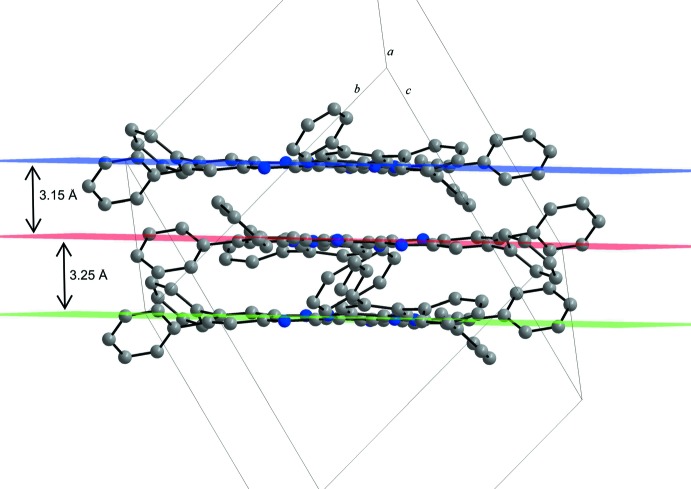
View along the plane defined by the central ring of HATNPh_6_ mol­ecules showing π–π inter­actions of the parallel displaced mol­ecules. H atoms and solvent mol­ecules are omitted for clarity. Colour code: C grey, N blue spheres.

**Figure 6:**
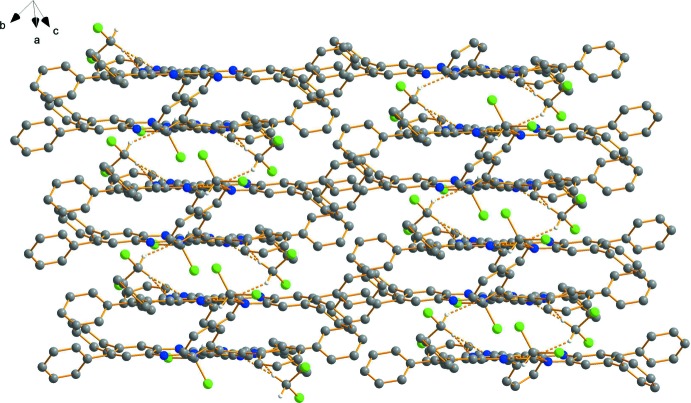
Packing diagram of HATNPh_6_ (**2**) viewed along the plane defined by the central ring of the mol­ecules. H atoms have been omitted for clarity. Dashed lines represent hydrogen bonds. Colour code: C grey, N blue, Cl green spheres.

The mol­ecules of HATNPh_6_ (**2**) form centrosymmetric dimers that are stacked perfectly parallel by van der Waals inter­actions but with a parallel displaced π-stacking. The plane-to-plane distance (defined by the central rings) within a dimer of 3.2518 (5) Å is shorter compared to the corres­ponding distance in **1**. This distance, as well as the short centroid-to-centroid distance of 3.4018 (7) Å are both at the lower limit of ranges known for metal complexes with aromatic nitro­gen-containing ligands (Janiak, 2000 ▸). The plane-to-plane distance between adjacent dimers is 3.15 Å. The parallel displacement between the layers (Fig. 5 ▸) is much shorter than for HATNMe_6_ (**1**), with an angle of 16.8° and a shift of approximately 1 Å. Comparing the plane-to-plane distances of the title compounds with related derivatives like HATN (Alfonso & Stoeckli-Evans, 2001 ▸; 3.66 Å) and HAT(CONH_2_)_6_ (Beeson *et al.*, 1996 ▸; 3.31 Å), the dimers of HATNPh_6_ (**2**) have the shortest contact and the shortest displacement in π-stacking. Further inter­actions between the terminal phenyl rings and the pyrazines rings inter­connect the dimers. The di­chloro­methane solvent mol­ecules are located near the electron lone pairs of the N atoms in the voids of the packed mol­ecules. They bridge two mol­ecules of **2** and consolidate the crystal packing through weak C—H⋯N hydrogen-bonding inter­actions (Table 1 ▸, Fig. 6 ▸).

## Synthesis and crystallization   

Hexaketo­cyclo­hexane octa­hydrate and 4,5-diphenyl-1,2-di­amine were prepared according to published procedures (Fatiadi & Sager, 1962 ▸; Shao *et al.*, 2012 ▸; Gao *et al.*, 2009 ▸).

**Synthesis of 1.** HATNMe_6_ was synthesized by a published procedure (Catalano *et al.*, 1994 ▸). Crystals suitable for single crystal X-ray diffraction were obtained by slow evaporation of a benzene solution of **1**.

**Synthesis of 2.** HATNPh_6_ was synthesized based on a literature method (Gao *et al.*, 2009 ▸). 4,5-diphenyl-1,2-di­amine (1.8 g, 6.9 mmol) and hexa­keto­cyclo­hexane octa­hydrate (0.54 g, 1.72 mmol) in 100 ml acetic acid were heated up to 373 K for 36 h under a nitro­gen atmosphere. After cooling to room temperature the reaction mixture was filtrated and the resulting yellow solid was washed with plenty of water and 2 *M* KOH solution. The solid was suspended in a mixture of di­chloro­methane (100 ml) and a saturated K_2_CO_3_ solution (100 ml) overnight in order to remove all traces of acetic acid. After filtration and washing with water, the solid was dried in a vacuum to give **2** as a yellow solid in 72% yield. Crystals suitable for single crystal X-ray diffraction were obtained by slow evaporation of a CH_2_Cl_2_ solution of **2**.

## Refinement   

Crystal data, data collection and structure refinement details are summarized in Table 2 ▸. Hydrogen atoms bound to C atoms were located from difference-Fourier maps but were subsequently fixed to idealized positions using appropriate riding models.

## Supplementary Material

Crystal structure: contains datablock(s) 1, 2. DOI: 10.1107/S2056989018000725/wm5431sup1.cif

Structure factors: contains datablock(s) 1. DOI: 10.1107/S2056989018000725/wm54311sup2.hkl

Structure factors: contains datablock(s) 2. DOI: 10.1107/S2056989018000725/wm54312sup3.hkl

Supporting information file. DOI: 10.1107/S2056989018000725/wm54311sup4.cml

Supporting information file. DOI: 10.1107/S2056989018000725/wm54312sup5.cml

CCDC references: 1816408, 1816407

Additional supporting information:  crystallographic information; 3D view; checkCIF report

## Figures and Tables

**Figure 4:**
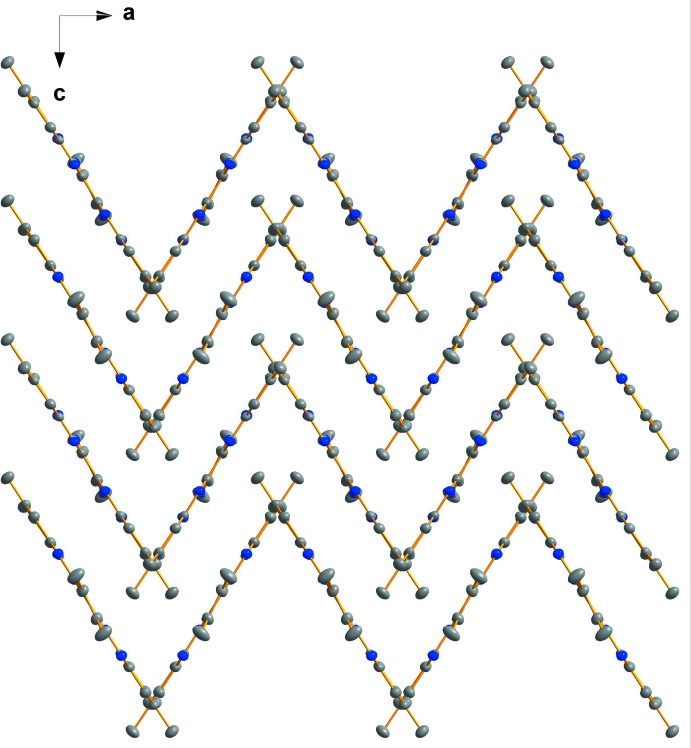
View along the *b* axis showing the packing of HATNMe_6_ (**1**) in a herringbone-like arrangement. H atoms have been omitted for clarity. Colour code: C grey, N blue spheres.

**Table 1:** Hydrogen-bond geometry (Å, °) for **2** [Chem scheme1]

*D*—H⋯*A*	*D*—H	H⋯*A*	*D*⋯*A*	*D*—H⋯*A*
C61—H61*A*⋯N1^i^	0.99	2.46	3.2380 (17)	135
C61—H61*B*⋯N2	0.99	2.40	3.2745 (17)	146
C61—H61*B*⋯N3	0.99	2.61	3.4923 (18)	149
C62—H62*A*⋯N4	0.99	2.58	3.2547 (17)	126
C62—H62*A*⋯N5	0.99	2.46	3.4381 (17)	169

Symmetry code: (i) 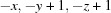.

**Table 2:** Experimental details

	**1**	**2**
Crystal data		
Chemical formula	C_30_H_24_N_6_	C_60_H_36_N_6_·2CH_2_Cl_2_
*M*_r_	468.55	1010.80
Crystal system, space group	Orthorhombic, *P* *b* *c* *n*	Triclinic, *P* 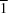
Temperature (K)	153	100
*a*, *b*, *c* (Å)	11.6178 (8), 15.7762 (8), 12.8621 (7)	9.2629 (4), 16.3829 (6), 18.4366 (6)
α, β, γ (°)	90, 90, 90	64.2659 (13), 78.2616 (15), 88.3530 (17)
*V* (Å^3^)	2357.4 (2)	2461.98 (16)
*Z*	4	2
Radiation type	Mo *K*α	Mo *K*α
μ (mm^−1^)	0.08	0.29
Crystal size (mm)	0.50 × 0.38 × 0.25	0.30 × 0.12 × 0.10

Data collection		
Diffractometer	Stoe IPDS	Bruker APEXII CCD
Absorption correction	–	Multi-scan (*SADABS*; Krause *et al.*, 2015 ▸)
*T*_min_, *T*_max_	–	0.970, 1.000
No. of measured, independent and observed [*I* > 2σ(*I*)] reflections	23121, 2361, 1286	87137, 14377, 11804
*R*_int_	0.057	0.043
(sin θ/λ)_max_ (Å^−1^)	0.621	0.704

Refinement		
*R*[*F* ^2^ > 2σ(*F* ^2^)], *wR*(*F* ^2^), *S*	0.032, 0.080, 0.75	0.039, 0.107, 1.02
No. of reflections	2361	14377
No. of parameters	166	649
H-atom treatment	H-atom parameters constrained	H-atom parameters constrained
Δρ_max_, Δρ_min_ (e Å^−3^)	0.16, −0.15	0.84, −0.84

Computer programs: *IPDS* (Stoe, 1999 ▸), *APEX2* and *SAINT* (Bruker, 2013 ▸), *X-RED* (Stoe, 2002 ▸), *SHELXS97* (Sheldrick, 2008 ▸), *SHELXT2013/1* (Sheldrick, 2015*a* ▸), *SHELXL2017/1* (Sheldrick, 2015*b* ▸), *DIAMOND* (Brandenburg & Putz, 2006 ▸) and *publCIF* (Westrip, 2010 ▸).

## supplementary crystallographic information

### 2,3,8,9,14,15-Hexamethyl-5,6,11,12,17,18-hexaazatrinaphthylene (1) Crystal data

**Table:**

C_30_H_24_N_6_	*D*_x_ = 1.320 Mg m^−^^3^
*M**_r_* = 468.55	Mo *K*α radiation, λ = 0.71073 Å
Orthorhombic, *P**b**c**n*	Cell parameters from 5493 reflections
*a* = 11.6178 (8) Å	θ = 2.3–26.2°
*b* = 15.7762 (8) Å	µ = 0.08 mm^−^^1^
*c* = 12.8621 (7) Å	*T* = 153 K
*V* = 2357.4 (2) Å^3^	Prism, yellow
*Z* = 4	0.50 × 0.38 × 0.25 mm
*F*(000) = 984	

### 2,3,8,9,14,15-Hexamethyl-5,6,11,12,17,18-hexaazatrinaphthylene (1) Data collection

**Table:**

Stoe IPDS diffractometer	*R*_int_ = 0.057
Radiation source: sealed tube	θ_max_ = 26.2°, θ_min_ = 2.6°
φ scans	*h* = −14→14
23121 measured reflections	*k* = −19→19
2361 independent reflections	*l* = −15→16
1286 reflections with *I* > 2σ(*I*)	

### 2,3,8,9,14,15-Hexamethyl-5,6,11,12,17,18-hexaazatrinaphthylene (1) Refinement

**Table:**

Refinement on *F*^2^	0 restraints
Least-squares matrix: full	Hydrogen site location: inferred from neighbouring sites
*R*[*F*^2^ > 2σ(*F*^2^)] = 0.032	H-atom parameters constrained
*wR*(*F*^2^) = 0.080	*w* = 1/[σ^2^(*F*_o_^2^) + (0.050*P*)^2^] where *P* = (*F*_o_^2^ + 2*F*_c_^2^)/3
*S* = 0.75	(Δ/σ)_max_ < 0.001
2361 reflections	Δρ_max_ = 0.16 e Å^−^^3^
166 parameters	Δρ_min_ = −0.14 e Å^−^^3^

### 2,3,8,9,14,15-Hexamethyl-5,6,11,12,17,18-hexaazatrinaphthylene (1) Special details

**Table:**

Geometry. All esds (except the esd in the dihedral angle between two l.s. planes) are estimated using the full covariance matrix. The cell esds are taken into account individually in the estimation of esds in distances, angles and torsion angles; correlations between esds in cell parameters are only used when they are defined by crystal symmetry. An approximate (isotropic) treatment of cell esds is used for estimating esds involving l.s. planes.

### 2,3,8,9,14,15-Hexamethyl-5,6,11,12,17,18-hexaazatrinaphthylene (1) Fractional atomic coordinates and isotropic or equivalent isotropic displacement parameters (Å^2^)

**Table:**

	*x*	*y*	*z*	*U*_iso_*/*U*_eq_	
N1	0.56074 (10)	0.64519 (8)	0.34566 (8)	0.0255 (3)	
N2	0.62730 (10)	0.49478 (7)	0.43437 (8)	0.0265 (3)	
N3	0.56076 (10)	0.34059 (8)	0.33894 (7)	0.0252 (3)	
C1	0.53175 (13)	0.57382 (8)	0.29813 (10)	0.0226 (3)	
C2	0.56581 (11)	0.49314 (9)	0.34649 (9)	0.0225 (3)	
C3	0.53343 (12)	0.41546 (9)	0.29893 (9)	0.0225 (3)	
C4	0.52959 (12)	0.71863 (9)	0.29846 (10)	0.0247 (3)	
C5	0.55513 (12)	0.79701 (10)	0.34611 (10)	0.0283 (3)	
H5	0.592691	0.797343	0.411715	0.034*	
C6	0.52734 (13)	0.87212 (9)	0.30047 (11)	0.0314 (4)	
C7	0.55470 (17)	0.95456 (11)	0.35433 (13)	0.0502 (5)	
H7A	0.579276	0.943076	0.425845	0.075*	
H7B	0.485952	0.990540	0.355234	0.075*	
H7C	0.616673	0.983645	0.316924	0.075*	
C8	0.65625 (12)	0.41882 (9)	0.47584 (10)	0.0246 (3)	
C9	0.72310 (13)	0.41486 (9)	0.56818 (10)	0.0288 (4)	
H9	0.745094	0.466061	0.601600	0.035*	
C10	0.75658 (12)	0.33909 (9)	0.61015 (10)	0.0281 (3)	
C11	0.72339 (12)	0.26111 (9)	0.56134 (10)	0.0266 (3)	
C12	0.65713 (13)	0.26394 (9)	0.47327 (10)	0.0269 (3)	
H12	0.633497	0.212331	0.441761	0.032*	
C13	0.62308 (11)	0.34154 (9)	0.42814 (9)	0.0238 (3)	
C14	0.82859 (14)	0.33631 (11)	0.70797 (11)	0.0398 (4)	
H14A	0.846997	0.394234	0.729895	0.060*	
H14B	0.900037	0.305244	0.694236	0.060*	
H14C	0.785419	0.307648	0.763184	0.060*	
C15	0.76172 (14)	0.17752 (9)	0.60460 (11)	0.0367 (4)	
H15A	0.729041	0.131482	0.562831	0.055*	
H15B	0.735262	0.172194	0.676654	0.055*	
H15C	0.845931	0.174242	0.602613	0.055*	

### 2,3,8,9,14,15-Hexamethyl-5,6,11,12,17,18-hexaazatrinaphthylene (1) Atomic displacement parameters (Å^2^)

**Table:**

	*U*^11^	*U*^22^	*U*^33^	*U*^12^	*U*^13^	*U*^23^
N1	0.0268 (6)	0.0259 (7)	0.0237 (5)	−0.0006 (6)	−0.0010 (5)	0.0007 (5)
N2	0.0279 (6)	0.0280 (7)	0.0235 (6)	−0.0014 (6)	−0.0026 (5)	0.0015 (5)
N3	0.0279 (6)	0.0277 (7)	0.0201 (5)	0.0003 (6)	−0.0001 (5)	0.0010 (5)
C1	0.0211 (7)	0.0255 (9)	0.0213 (6)	−0.0007 (6)	0.0018 (6)	−0.0005 (5)
C2	0.0209 (7)	0.0265 (8)	0.0201 (6)	0.0010 (7)	0.0028 (6)	0.0001 (6)
C3	0.0224 (7)	0.0259 (8)	0.0191 (6)	0.0008 (6)	0.0034 (6)	0.0006 (5)
C4	0.0225 (7)	0.0271 (9)	0.0244 (7)	−0.0003 (7)	0.0010 (6)	0.0011 (6)
C5	0.0274 (8)	0.0313 (9)	0.0262 (7)	−0.0004 (7)	−0.0058 (6)	−0.0017 (6)
C6	0.0311 (8)	0.0272 (9)	0.0360 (8)	−0.0017 (7)	−0.0061 (6)	−0.0015 (6)
C7	0.0685 (13)	0.0294 (10)	0.0526 (9)	0.0004 (9)	−0.0288 (9)	−0.0037 (8)
C8	0.0236 (8)	0.0277 (9)	0.0225 (7)	0.0009 (6)	0.0014 (6)	0.0025 (6)
C9	0.0311 (9)	0.0301 (9)	0.0254 (7)	−0.0031 (7)	−0.0033 (6)	−0.0006 (6)
C10	0.0267 (8)	0.0323 (9)	0.0253 (6)	−0.0006 (7)	−0.0011 (6)	0.0061 (6)
C11	0.0261 (8)	0.0287 (9)	0.0252 (7)	0.0031 (6)	0.0044 (6)	0.0049 (6)
C12	0.0306 (8)	0.0257 (8)	0.0246 (7)	0.0003 (6)	0.0025 (6)	−0.0011 (5)
C13	0.0238 (7)	0.0277 (8)	0.0198 (6)	0.0012 (7)	0.0027 (5)	0.0010 (6)
C14	0.0426 (9)	0.0399 (10)	0.0370 (8)	−0.0030 (8)	−0.0141 (7)	0.0061 (7)
C15	0.0385 (10)	0.0365 (10)	0.0350 (8)	0.0033 (8)	−0.0042 (7)	0.0063 (6)

### 2,3,8,9,14,15-Hexamethyl-5,6,11,12,17,18-hexaazatrinaphthylene (1) Geometric parameters (Å, º)

**Table:**

N1—C1	1.3248 (17)	C7—H7C	0.9800
N1—C4	1.3571 (18)	C8—C13	1.4181 (19)
N2—C2	1.3374 (15)	C8—C9	1.4205 (19)
N2—C8	1.3541 (18)	C9—C10	1.368 (2)
N3—C3	1.3270 (17)	C9—H9	0.9500
N3—C13	1.3568 (16)	C10—C11	1.434 (2)
C1—C1^i^	1.441 (3)	C10—C14	1.5115 (19)
C1—C2	1.4709 (19)	C11—C12	1.3703 (19)
C2—C3	1.4203 (19)	C11—C15	1.4990 (19)
C3—C3^i^	1.479 (3)	C12—C13	1.4115 (19)
C4—C5	1.412 (2)	C12—H12	0.9500
C4—C4^i^	1.424 (3)	C14—H14A	0.9800
C5—C6	1.361 (2)	C14—H14B	0.9800
C5—H5	0.9500	C14—H14C	0.9800
C6—C6^i^	1.445 (3)	C15—H15A	0.9800
C6—C7	1.507 (2)	C15—H15B	0.9800
C7—H7A	0.9800	C15—H15C	0.9800
C7—H7B	0.9800		
			
C1—N1—C4	116.83 (11)	C13—C8—C9	118.20 (12)
C2—N2—C8	116.65 (12)	C10—C9—C8	121.58 (14)
C3—N3—C13	116.47 (12)	C10—C9—H9	119.2
N1—C1—C1^i^	121.77 (8)	C8—C9—H9	119.2
N1—C1—C2	118.16 (12)	C9—C10—C11	120.03 (12)
C1^i^—C1—C2	120.07 (7)	C9—C10—C14	120.74 (14)
N2—C2—C3	121.48 (13)	C11—C10—C14	119.24 (13)
N2—C2—C1	118.97 (13)	C12—C11—C10	119.02 (13)
C3—C2—C1	119.55 (11)	C12—C11—C15	120.17 (13)
N3—C3—C2	122.52 (12)	C10—C11—C15	120.81 (12)
N3—C3—C3^i^	117.11 (7)	C11—C12—C13	121.71 (13)
C2—C3—C3^i^	120.37 (8)	C11—C12—H12	119.1
N1—C4—C5	119.83 (12)	C13—C12—H12	119.1
N1—C4—C4^i^	121.37 (7)	N3—C13—C12	119.18 (13)
C5—C4—C4^i^	118.80 (8)	N3—C13—C8	121.36 (12)
C6—C5—C4	121.70 (12)	C12—C13—C8	119.45 (11)
C6—C5—H5	119.1	C10—C14—H14A	109.5
C4—C5—H5	119.1	C10—C14—H14B	109.5
C5—C6—C6^i^	119.44 (8)	H14A—C14—H14B	109.5
C5—C6—C7	120.19 (13)	C10—C14—H14C	109.5
C6^i^—C6—C7	120.36 (9)	H14A—C14—H14C	109.5
C6—C7—H7A	109.5	H14B—C14—H14C	109.5
C6—C7—H7B	109.5	C11—C15—H15A	109.5
H7A—C7—H7B	109.5	C11—C15—H15B	109.5
C6—C7—H7C	109.5	H15A—C15—H15B	109.5
H7A—C7—H7C	109.5	C11—C15—H15C	109.5
H7B—C7—H7C	109.5	H15A—C15—H15C	109.5
N2—C8—C13	121.52 (12)	H15B—C15—H15C	109.5
N2—C8—C9	120.27 (13)		
			
C4—N1—C1—C1^i^	−1.1 (2)	C2—N2—C8—C13	0.12 (18)
C4—N1—C1—C2	179.08 (13)	C2—N2—C8—C9	178.97 (12)
C8—N2—C2—C3	−0.44 (17)	N2—C8—C9—C10	−178.02 (13)
C8—N2—C2—C1	179.87 (12)	C13—C8—C9—C10	0.9 (2)
N1—C1—C2—N2	−1.96 (18)	C8—C9—C10—C11	−0.5 (2)
C1^i^—C1—C2—N2	178.26 (15)	C8—C9—C10—C14	179.46 (14)
N1—C1—C2—C3	178.35 (13)	C9—C10—C11—C12	−0.7 (2)
C1^i^—C1—C2—C3	−1.4 (2)	C14—C10—C11—C12	179.35 (13)
C13—N3—C3—C2	−0.70 (18)	C9—C10—C11—C15	178.43 (14)
C13—N3—C3—C3^i^	179.36 (15)	C14—C10—C11—C15	−1.56 (19)
N2—C2—C3—N3	0.77 (19)	C10—C11—C12—C13	1.5 (2)
C1—C2—C3—N3	−179.54 (13)	C15—C11—C12—C13	−177.59 (13)
N2—C2—C3—C3^i^	−179.28 (15)	C3—N3—C13—C12	−178.55 (12)
C1—C2—C3—C3^i^	0.4 (2)	C3—N3—C13—C8	0.36 (17)
C1—N1—C4—C5	178.39 (14)	C11—C12—C13—N3	177.78 (13)
C1—N1—C4—C4^i^	−1.1 (2)	C11—C12—C13—C8	−1.2 (2)
N1—C4—C5—C6	178.74 (15)	N2—C8—C13—N3	−0.1 (2)
C4^i^—C4—C5—C6	−1.7 (2)	C9—C8—C13—N3	−178.96 (13)
C4—C5—C6—C6^i^	−1.5 (3)	N2—C8—C13—C12	178.83 (14)
C4—C5—C6—C7	179.25 (15)	C9—C8—C13—C12	−0.04 (18)

Symmetry code: (i) −*x*+1, *y*, −*z*+1/2.

### 2,3,8,9,14,15-Hexaphenyl-5,6,11,12,17,18-hexazatrinaphthylene dichloromethane disolvate (2) Crystal data

**Table:**

C_60_H_36_N_6_·2CH_2_Cl_2_	*Z* = 2
*M**_r_* = 1010.80	*F*(000) = 1044
Triclinic, *P*1	*D*_x_ = 1.364 Mg m^−^^3^
*a* = 9.2629 (4) Å	Mo *K*α radiation, λ = 0.71073 Å
*b* = 16.3829 (6) Å	Cell parameters from 9893 reflections
*c* = 18.4366 (6) Å	θ = 2.3–30.0°
α = 64.2659 (13)°	µ = 0.29 mm^−^^1^
β = 78.2616 (15)°	*T* = 100 K
γ = 88.3530 (17)°	Block, yellow
*V* = 2461.98 (16) Å^3^	0.30 × 0.12 × 0.10 mm

### 2,3,8,9,14,15-Hexaphenyl-5,6,11,12,17,18-hexazatrinaphthylene dichloromethane disolvate (2) Data collection

**Table:**

Bruker APEX-II CCD diffractometer	11804 reflections with *I* > 2σ(*I*)
Radiation source: sealed tube	*R*_int_ = 0.043
φ and ω scans	θ_max_ = 30.0°, θ_min_ = 1.4°
Absorption correction: multi-scan (*SADABS*; Krause *et al.*, 2015)	*h* = −13→13
*T*_min_ = 0.970, *T*_max_ = 1.000	*k* = −23→23
87137 measured reflections	*l* = −25→25
14377 independent reflections	

### 2,3,8,9,14,15-Hexaphenyl-5,6,11,12,17,18-hexazatrinaphthylene dichloromethane disolvate (2) Refinement

**Table:**

Refinement on *F*^2^	Primary atom site location: structure-invariant direct methods
Least-squares matrix: full	Secondary atom site location: difference Fourier map
*R*[*F*^2^ > 2σ(*F*^2^)] = 0.039	Hydrogen site location: difference Fourier map
*wR*(*F*^2^) = 0.107	H-atom parameters constrained
*S* = 1.02	*w* = 1/[σ^2^(*F*_o_^2^) + (0.050*P*)^2^ + 1.2*P*] where *P* = (*F*_o_^2^ + 2*F*_c_^2^)/3
14377 reflections	(Δ/σ)_max_ = 0.001
649 parameters	Δρ_max_ = 0.84 e Å^−^^3^
0 restraints	Δρ_min_ = −0.84 e Å^−^^3^

### 2,3,8,9,14,15-Hexaphenyl-5,6,11,12,17,18-hexazatrinaphthylene dichloromethane disolvate (2) Special details

**Table:**

Geometry. All esds (except the esd in the dihedral angle between two l.s. planes) are estimated using the full covariance matrix. The cell esds are taken into account individually in the estimation of esds in distances, angles and torsion angles; correlations between esds in cell parameters are only used when they are defined by crystal symmetry. An approximate (isotropic) treatment of cell esds is used for estimating esds involving l.s. planes.

### 2,3,8,9,14,15-Hexaphenyl-5,6,11,12,17,18-hexazatrinaphthylene dichloromethane disolvate (2) Fractional atomic coordinates and isotropic or equivalent isotropic displacement parameters (Å^2^)

**Table:**

	*x*	*y*	*z*	*U*_iso_*/*U*_eq_	
N1	0.11926 (11)	0.44967 (7)	0.60814 (6)	0.01193 (18)	
N2	0.18060 (11)	0.60400 (7)	0.45277 (6)	0.01252 (19)	
N3	0.41523 (11)	0.61360 (7)	0.33603 (6)	0.01264 (19)	
N4	0.59471 (11)	0.46401 (7)	0.36209 (6)	0.01232 (19)	
N5	0.53889 (11)	0.31547 (7)	0.51241 (6)	0.01196 (18)	
N6	0.27642 (11)	0.29872 (7)	0.62955 (6)	0.01280 (19)	
C1	0.22651 (12)	0.45307 (8)	0.54672 (7)	0.0107 (2)	
C2	0.25958 (12)	0.53194 (8)	0.46923 (7)	0.0111 (2)	
C3	0.38735 (12)	0.53672 (8)	0.40497 (7)	0.0110 (2)	
C4	0.47903 (12)	0.46219 (8)	0.41903 (7)	0.0109 (2)	
C5	0.44562 (13)	0.38091 (8)	0.49903 (7)	0.0110 (2)	
C6	0.31672 (12)	0.37406 (8)	0.55998 (7)	0.0113 (2)	
C7	0.04205 (13)	0.52505 (8)	0.59315 (7)	0.0116 (2)	
C8	−0.06780 (13)	0.53019 (8)	0.65653 (7)	0.0132 (2)	
H8	−0.088884	0.479364	0.709104	0.016*	
C9	−0.14549 (13)	0.60696 (8)	0.64431 (7)	0.0128 (2)	
C10	−0.12054 (13)	0.68304 (8)	0.56292 (7)	0.0128 (2)	
C11	−0.01485 (13)	0.67705 (8)	0.50117 (7)	0.0142 (2)	
H11	0.000554	0.725739	0.447369	0.017*	
C12	0.07124 (13)	0.60140 (8)	0.51484 (7)	0.0122 (2)	
C13	−0.24727 (13)	0.60780 (8)	0.71806 (7)	0.0140 (2)	
C14	−0.34319 (14)	0.53190 (9)	0.77210 (8)	0.0175 (2)	
H14	−0.344709	0.480483	0.761037	0.021*	
C15	−0.43664 (15)	0.53104 (10)	0.84210 (8)	0.0229 (3)	
H15	−0.502156	0.479426	0.878182	0.028*	
C16	−0.43391 (16)	0.60558 (11)	0.85904 (9)	0.0244 (3)	
H16	−0.497906	0.605196	0.906536	0.029*	
C17	−0.33734 (16)	0.68085 (10)	0.80636 (9)	0.0233 (3)	
H17	−0.334699	0.731629	0.818261	0.028*	
C18	−0.24461 (15)	0.68200 (9)	0.73633 (8)	0.0181 (2)	
H18	−0.178930	0.733655	0.700611	0.022*	
C19	−0.20291 (13)	0.76731 (8)	0.53886 (7)	0.0139 (2)	
C20	−0.35316 (14)	0.76736 (9)	0.57128 (8)	0.0161 (2)	
H20	−0.404730	0.713178	0.614174	0.019*	
C21	−0.42778 (14)	0.84646 (9)	0.54103 (8)	0.0185 (2)	
H21	−0.529303	0.845922	0.564415	0.022*	
C22	−0.35570 (15)	0.92581 (9)	0.47732 (8)	0.0197 (3)	
H22	−0.408316	0.978749	0.455846	0.024*	
C23	−0.20617 (15)	0.92744 (9)	0.44508 (8)	0.0193 (2)	
H23	−0.155738	0.981790	0.401845	0.023*	
C24	−0.13024 (14)	0.84933 (9)	0.47618 (8)	0.0172 (2)	
H24	−0.027381	0.851395	0.454697	0.021*	
C25	0.53512 (13)	0.61711 (8)	0.27859 (7)	0.0127 (2)	
C26	0.57330 (13)	0.69798 (8)	0.20488 (7)	0.0144 (2)	
H26	0.516697	0.749110	0.198040	0.017*	
C27	0.69015 (13)	0.70462 (8)	0.14283 (7)	0.0134 (2)	
C28	0.77379 (13)	0.62586 (8)	0.15212 (7)	0.0136 (2)	
C29	0.74119 (13)	0.54812 (8)	0.22552 (7)	0.0145 (2)	
H29	0.799147	0.497494	0.232495	0.017*	
C30	0.62378 (13)	0.54171 (8)	0.29071 (7)	0.0124 (2)	
C31	0.72725 (13)	0.79506 (8)	0.07089 (7)	0.0135 (2)	
C32	0.61387 (14)	0.84510 (9)	0.03578 (8)	0.0161 (2)	
H32	0.515374	0.818781	0.054791	0.019*	
C33	0.64453 (15)	0.93319 (9)	−0.02681 (8)	0.0183 (2)	
H33	0.566824	0.966807	−0.050029	0.022*	
C34	0.78795 (15)	0.97199 (9)	−0.05534 (8)	0.0184 (2)	
H34	0.808594	1.032244	−0.097779	0.022*	
C35	0.90129 (15)	0.92247 (9)	−0.02165 (8)	0.0186 (2)	
H35	0.999785	0.948852	−0.041568	0.022*	
C36	0.87189 (14)	0.83442 (9)	0.04111 (8)	0.0168 (2)	
H36	0.950247	0.801027	0.063748	0.020*	
C37	0.88799 (14)	0.62319 (8)	0.08340 (7)	0.0145 (2)	
C38	0.86264 (15)	0.65702 (9)	0.00340 (8)	0.0192 (2)	
H38	0.774443	0.686153	−0.008436	0.023*	
C39	0.96540 (16)	0.64836 (10)	−0.05883 (8)	0.0222 (3)	
H39	0.946888	0.671517	−0.112837	0.027*	
C40	1.09477 (16)	0.60612 (9)	−0.04259 (9)	0.0226 (3)	
H40	1.164511	0.600156	−0.085274	0.027*	
C41	1.12205 (15)	0.57251 (9)	0.03644 (9)	0.0206 (3)	
H41	1.210638	0.543670	0.047843	0.025*	
C42	1.01925 (14)	0.58124 (9)	0.09874 (8)	0.0172 (2)	
H42	1.038596	0.558314	0.152556	0.021*	
C43	0.50237 (13)	0.23986 (8)	0.58462 (7)	0.0120 (2)	
C44	0.59874 (13)	0.16847 (8)	0.60277 (7)	0.0136 (2)	
H44	0.691680	0.176693	0.566382	0.016*	
C45	0.56063 (13)	0.08732 (8)	0.67207 (7)	0.0127 (2)	
C46	0.41889 (13)	0.07453 (8)	0.72707 (7)	0.0132 (2)	
C47	0.32768 (13)	0.14531 (8)	0.71244 (7)	0.0142 (2)	
H47	0.236793	0.137546	0.750318	0.017*	
C48	0.36715 (13)	0.22959 (8)	0.64178 (7)	0.0126 (2)	
C49	0.67019 (13)	0.01680 (8)	0.68874 (7)	0.0133 (2)	
C50	0.74324 (14)	−0.00492 (9)	0.62584 (8)	0.0165 (2)	
H50	0.717486	0.022263	0.573447	0.020*	
C51	0.85324 (15)	−0.06598 (9)	0.63947 (8)	0.0197 (3)	
H51	0.901409	−0.080790	0.596500	0.024*	
C52	0.89332 (15)	−0.10554 (9)	0.71539 (8)	0.0198 (3)	
H52	0.970025	−0.146296	0.724094	0.024*	
C53	0.82030 (15)	−0.08504 (9)	0.77862 (8)	0.0189 (2)	
H53	0.846503	−0.112295	0.830896	0.023*	
C54	0.70939 (14)	−0.02491 (9)	0.76543 (8)	0.0162 (2)	
H54	0.659286	−0.011860	0.809141	0.019*	
C55	0.36807 (13)	−0.01519 (8)	0.79842 (7)	0.0143 (2)	
C56	0.36616 (16)	−0.09418 (9)	0.78719 (8)	0.0212 (3)	
H56	0.397645	−0.090807	0.733542	0.025*	
C57	0.31856 (18)	−0.17757 (9)	0.85392 (9)	0.0259 (3)	
H57	0.316230	−0.230743	0.845538	0.031*	
C58	0.27434 (16)	−0.18366 (9)	0.93285 (9)	0.0226 (3)	
H58	0.243054	−0.240893	0.978501	0.027*	
C59	0.27614 (15)	−0.10594 (9)	0.94456 (8)	0.0184 (2)	
H59	0.246541	−0.109943	0.998515	0.022*	
C60	0.32114 (14)	−0.02160 (8)	0.87767 (8)	0.0153 (2)	
H60	0.319801	0.031642	0.886143	0.018*	
Cl1	0.11062 (5)	0.83590 (3)	0.26669 (2)	0.03512 (10)	
Cl2	0.17654 (4)	0.72871 (3)	0.17281 (2)	0.02962 (9)	
C61	0.10609 (16)	0.72602 (9)	0.27127 (8)	0.0218 (3)	
H61A	0.002980	0.699304	0.291784	0.026*	
H61B	0.165617	0.686843	0.310597	0.026*	
Cl3	0.70558 (5)	0.30628 (3)	0.27367 (2)	0.03072 (9)	
Cl4	0.84801 (4)	0.18745 (2)	0.40649 (2)	0.02374 (8)	
C62	0.78838 (16)	0.29773 (9)	0.35492 (9)	0.0208 (3)	
H62A	0.716264	0.311995	0.394731	0.025*	
H62B	0.874064	0.342747	0.332468	0.025*	

### 2,3,8,9,14,15-Hexaphenyl-5,6,11,12,17,18-hexazatrinaphthylene dichloromethane disolvate (2) Atomic displacement parameters (Å^2^)

**Table:**

	*U*^11^	*U*^22^	*U*^33^	*U*^12^	*U*^13^	*U*^23^
N1	0.0112 (4)	0.0113 (4)	0.0130 (4)	0.0012 (4)	−0.0023 (4)	−0.0051 (4)
N2	0.0130 (4)	0.0120 (5)	0.0124 (4)	0.0027 (4)	−0.0030 (4)	−0.0051 (4)
N3	0.0135 (5)	0.0116 (5)	0.0116 (4)	0.0015 (4)	−0.0019 (4)	−0.0044 (4)
N4	0.0124 (4)	0.0119 (5)	0.0112 (4)	0.0011 (4)	−0.0016 (4)	−0.0041 (4)
N5	0.0127 (4)	0.0112 (5)	0.0111 (4)	0.0017 (4)	−0.0029 (4)	−0.0040 (4)
N6	0.0127 (4)	0.0119 (5)	0.0120 (4)	0.0020 (4)	−0.0020 (4)	−0.0040 (4)
C1	0.0102 (5)	0.0105 (5)	0.0117 (5)	0.0010 (4)	−0.0033 (4)	−0.0046 (4)
C2	0.0110 (5)	0.0108 (5)	0.0114 (5)	0.0011 (4)	−0.0026 (4)	−0.0047 (4)
C3	0.0106 (5)	0.0109 (5)	0.0110 (5)	0.0006 (4)	−0.0022 (4)	−0.0043 (4)
C4	0.0110 (5)	0.0107 (5)	0.0108 (5)	0.0008 (4)	−0.0025 (4)	−0.0043 (4)
C5	0.0112 (5)	0.0112 (5)	0.0102 (5)	0.0005 (4)	−0.0021 (4)	−0.0044 (4)
C6	0.0112 (5)	0.0110 (5)	0.0115 (5)	0.0013 (4)	−0.0031 (4)	−0.0046 (4)
C7	0.0113 (5)	0.0113 (5)	0.0122 (5)	0.0009 (4)	−0.0024 (4)	−0.0051 (4)
C8	0.0128 (5)	0.0128 (5)	0.0127 (5)	0.0003 (4)	−0.0015 (4)	−0.0048 (4)
C9	0.0111 (5)	0.0142 (5)	0.0135 (5)	0.0004 (4)	−0.0018 (4)	−0.0070 (4)
C10	0.0122 (5)	0.0120 (5)	0.0150 (5)	0.0021 (4)	−0.0034 (4)	−0.0064 (4)
C11	0.0150 (5)	0.0128 (5)	0.0132 (5)	0.0034 (4)	−0.0035 (4)	−0.0042 (4)
C12	0.0122 (5)	0.0121 (5)	0.0121 (5)	0.0012 (4)	−0.0032 (4)	−0.0049 (4)
C13	0.0123 (5)	0.0162 (6)	0.0142 (5)	0.0032 (4)	−0.0038 (4)	−0.0069 (5)
C14	0.0157 (6)	0.0183 (6)	0.0179 (6)	0.0006 (5)	−0.0021 (5)	−0.0079 (5)
C15	0.0184 (6)	0.0267 (7)	0.0185 (6)	−0.0012 (5)	0.0012 (5)	−0.0072 (5)
C16	0.0208 (6)	0.0340 (8)	0.0176 (6)	0.0055 (6)	0.0006 (5)	−0.0129 (6)
C17	0.0267 (7)	0.0270 (7)	0.0212 (6)	0.0066 (6)	−0.0037 (5)	−0.0160 (6)
C18	0.0190 (6)	0.0181 (6)	0.0174 (6)	0.0019 (5)	−0.0021 (5)	−0.0089 (5)
C19	0.0152 (5)	0.0140 (5)	0.0146 (5)	0.0040 (4)	−0.0051 (4)	−0.0075 (5)
C20	0.0152 (5)	0.0163 (6)	0.0190 (6)	0.0027 (4)	−0.0039 (5)	−0.0098 (5)
C21	0.0140 (5)	0.0202 (6)	0.0252 (6)	0.0056 (5)	−0.0055 (5)	−0.0132 (5)
C22	0.0216 (6)	0.0173 (6)	0.0236 (6)	0.0089 (5)	−0.0095 (5)	−0.0104 (5)
C23	0.0228 (6)	0.0140 (6)	0.0185 (6)	0.0033 (5)	−0.0044 (5)	−0.0048 (5)
C24	0.0158 (6)	0.0175 (6)	0.0178 (6)	0.0037 (5)	−0.0022 (5)	−0.0080 (5)
C25	0.0127 (5)	0.0127 (5)	0.0120 (5)	0.0010 (4)	−0.0023 (4)	−0.0048 (4)
C26	0.0150 (5)	0.0117 (5)	0.0138 (5)	0.0025 (4)	−0.0031 (4)	−0.0032 (4)
C27	0.0135 (5)	0.0125 (5)	0.0121 (5)	0.0011 (4)	−0.0031 (4)	−0.0032 (4)
C28	0.0129 (5)	0.0142 (5)	0.0117 (5)	0.0007 (4)	−0.0015 (4)	−0.0044 (4)
C29	0.0142 (5)	0.0128 (5)	0.0139 (5)	0.0028 (4)	−0.0012 (4)	−0.0043 (4)
C30	0.0131 (5)	0.0114 (5)	0.0117 (5)	0.0010 (4)	−0.0026 (4)	−0.0041 (4)
C31	0.0153 (5)	0.0117 (5)	0.0108 (5)	0.0008 (4)	−0.0014 (4)	−0.0030 (4)
C32	0.0140 (5)	0.0176 (6)	0.0137 (5)	0.0018 (4)	−0.0018 (4)	−0.0046 (5)
C33	0.0222 (6)	0.0172 (6)	0.0135 (5)	0.0059 (5)	−0.0049 (5)	−0.0046 (5)
C34	0.0265 (7)	0.0135 (6)	0.0117 (5)	0.0006 (5)	−0.0018 (5)	−0.0033 (5)
C35	0.0179 (6)	0.0164 (6)	0.0171 (6)	−0.0037 (5)	0.0008 (5)	−0.0051 (5)
C36	0.0155 (6)	0.0153 (6)	0.0169 (6)	0.0012 (4)	−0.0032 (5)	−0.0047 (5)
C37	0.0158 (5)	0.0126 (5)	0.0122 (5)	−0.0013 (4)	0.0007 (4)	−0.0043 (4)
C38	0.0226 (6)	0.0170 (6)	0.0150 (6)	0.0012 (5)	−0.0023 (5)	−0.0052 (5)
C39	0.0304 (7)	0.0204 (6)	0.0127 (6)	−0.0022 (5)	0.0003 (5)	−0.0063 (5)
C40	0.0258 (7)	0.0195 (6)	0.0197 (6)	−0.0052 (5)	0.0071 (5)	−0.0108 (5)
C41	0.0176 (6)	0.0182 (6)	0.0236 (6)	−0.0008 (5)	0.0026 (5)	−0.0098 (5)
C42	0.0177 (6)	0.0158 (6)	0.0154 (6)	0.0005 (5)	−0.0008 (5)	−0.0054 (5)
C43	0.0136 (5)	0.0116 (5)	0.0104 (5)	0.0013 (4)	−0.0021 (4)	−0.0048 (4)
C44	0.0137 (5)	0.0138 (5)	0.0122 (5)	0.0032 (4)	−0.0017 (4)	−0.0052 (4)
C45	0.0148 (5)	0.0125 (5)	0.0112 (5)	0.0032 (4)	−0.0031 (4)	−0.0055 (4)
C46	0.0156 (5)	0.0116 (5)	0.0113 (5)	0.0012 (4)	−0.0027 (4)	−0.0041 (4)
C47	0.0143 (5)	0.0130 (5)	0.0122 (5)	0.0013 (4)	−0.0006 (4)	−0.0035 (4)
C48	0.0129 (5)	0.0120 (5)	0.0121 (5)	0.0014 (4)	−0.0025 (4)	−0.0046 (4)
C49	0.0143 (5)	0.0102 (5)	0.0134 (5)	0.0018 (4)	−0.0024 (4)	−0.0035 (4)
C50	0.0197 (6)	0.0153 (6)	0.0144 (5)	0.0038 (5)	−0.0033 (5)	−0.0067 (5)
C51	0.0227 (6)	0.0182 (6)	0.0196 (6)	0.0066 (5)	−0.0030 (5)	−0.0105 (5)
C52	0.0206 (6)	0.0162 (6)	0.0233 (6)	0.0073 (5)	−0.0063 (5)	−0.0090 (5)
C53	0.0228 (6)	0.0162 (6)	0.0172 (6)	0.0062 (5)	−0.0079 (5)	−0.0056 (5)
C54	0.0189 (6)	0.0151 (6)	0.0144 (5)	0.0044 (5)	−0.0035 (5)	−0.0065 (5)
C55	0.0143 (5)	0.0117 (5)	0.0135 (5)	0.0012 (4)	−0.0022 (4)	−0.0028 (4)
C56	0.0297 (7)	0.0156 (6)	0.0164 (6)	−0.0010 (5)	−0.0013 (5)	−0.0066 (5)
C57	0.0372 (8)	0.0130 (6)	0.0238 (7)	−0.0034 (6)	−0.0002 (6)	−0.0073 (5)
C58	0.0266 (7)	0.0133 (6)	0.0200 (6)	−0.0026 (5)	−0.0006 (5)	−0.0015 (5)
C59	0.0193 (6)	0.0169 (6)	0.0137 (5)	0.0003 (5)	0.0000 (5)	−0.0033 (5)
C60	0.0153 (5)	0.0136 (5)	0.0147 (5)	0.0012 (4)	−0.0015 (4)	−0.0048 (5)
Cl1	0.0569 (3)	0.01799 (17)	0.02317 (17)	−0.00789 (16)	−0.00341 (17)	−0.00389 (14)
Cl2	0.03465 (19)	0.0337 (2)	0.01836 (16)	0.00290 (15)	−0.00656 (14)	−0.00909 (14)
C61	0.0273 (7)	0.0158 (6)	0.0156 (6)	−0.0027 (5)	−0.0028 (5)	−0.0013 (5)
Cl3	0.0449 (2)	0.02792 (18)	0.01921 (16)	−0.00109 (16)	−0.01175 (15)	−0.00788 (14)
Cl4	0.02025 (15)	0.02240 (16)	0.02590 (16)	0.00880 (12)	−0.00300 (12)	−0.00941 (13)
C62	0.0233 (6)	0.0187 (6)	0.0233 (6)	0.0049 (5)	−0.0087 (5)	−0.0106 (5)

### 2,3,8,9,14,15-Hexaphenyl-5,6,11,12,17,18-hexazatrinaphthylene dichloromethane disolvate (2) Geometric parameters (Å, º)

**Table:**

N1—C1	1.3278 (15)	C31—C32	1.4001 (17)
N1—C7	1.3588 (15)	C32—C33	1.3925 (18)
N2—C2	1.3231 (15)	C32—H32	0.9500
N2—C12	1.3513 (15)	C33—C34	1.3854 (19)
N3—C3	1.3279 (15)	C33—H33	0.9500
N3—C25	1.3529 (15)	C34—C35	1.3875 (19)
N4—C4	1.3296 (15)	C34—H34	0.9500
N4—C30	1.3585 (15)	C35—C36	1.3923 (18)
N5—C5	1.3268 (15)	C35—H35	0.9500
N5—C43	1.3528 (15)	C36—H36	0.9500
N6—C6	1.3286 (15)	C37—C42	1.3978 (18)
N6—C48	1.3567 (15)	C37—C38	1.4004 (17)
C1—C2	1.4305 (16)	C38—C39	1.3899 (18)
C1—C6	1.4763 (16)	C38—H38	0.9500
C2—C3	1.4713 (16)	C39—C40	1.387 (2)
C3—C4	1.4263 (16)	C39—H39	0.9500
C4—C5	1.4755 (16)	C40—C41	1.391 (2)
C5—C6	1.4328 (16)	C40—H40	0.9500
C7—C8	1.4159 (16)	C41—C42	1.3915 (18)
C7—C12	1.4192 (16)	C41—H41	0.9500
C8—C9	1.3851 (16)	C42—H42	0.9500
C8—H8	0.9500	C43—C44	1.4136 (16)
C9—C10	1.4526 (17)	C43—C48	1.4244 (16)
C9—C13	1.4939 (16)	C44—C45	1.3759 (17)
C10—C11	1.3792 (16)	C44—H44	0.9500
C10—C19	1.4934 (16)	C45—C46	1.4440 (16)
C11—C12	1.4103 (16)	C45—C49	1.4868 (16)
C11—H11	0.9500	C46—C47	1.3770 (17)
C13—C18	1.3973 (17)	C46—C55	1.4905 (16)
C13—C14	1.3984 (18)	C47—C48	1.4179 (16)
C14—C15	1.3948 (18)	C47—H47	0.9500
C14—H14	0.9500	C49—C54	1.3977 (17)
C15—C16	1.388 (2)	C49—C50	1.3979 (17)
C15—H15	0.9500	C50—C51	1.3891 (18)
C16—C17	1.391 (2)	C50—H50	0.9500
C16—H16	0.9500	C51—C52	1.3880 (19)
C17—C18	1.3907 (18)	C51—H51	0.9500
C17—H17	0.9500	C52—C53	1.3911 (19)
C18—H18	0.9500	C52—H52	0.9500
C19—C20	1.3987 (17)	C53—C54	1.3858 (17)
C19—C24	1.4075 (18)	C53—H53	0.9500
C20—C21	1.3949 (17)	C54—H54	0.9500
C20—H20	0.9500	C55—C60	1.3949 (17)
C21—C22	1.386 (2)	C55—C56	1.3976 (18)
C21—H21	0.9500	C56—C57	1.3889 (19)
C22—C23	1.3877 (19)	C56—H56	0.9500
C22—H22	0.9500	C57—C58	1.389 (2)
C23—C24	1.3915 (18)	C57—H57	0.9500
C23—H23	0.9500	C58—C59	1.3818 (19)
C24—H24	0.9500	C58—H58	0.9500
C25—C26	1.4124 (16)	C59—C60	1.3957 (17)
C25—C30	1.4237 (16)	C59—H59	0.9500
C26—C27	1.3736 (17)	C60—H60	0.9500
C26—H26	0.9500	Cl1—C61	1.7665 (14)
C27—C28	1.4489 (17)	Cl2—C61	1.7812 (14)
C27—C31	1.4865 (16)	C61—H61A	0.9900
C28—C29	1.3798 (17)	C61—H61B	0.9900
C28—C37	1.4920 (16)	Cl3—C62	1.7695 (14)
C29—C30	1.4155 (16)	Cl4—C62	1.7713 (14)
C29—H29	0.9500	C62—H62A	0.9900
C31—C36	1.3983 (17)	C62—H62B	0.9900
			
C1—N1—C7	116.30 (10)	C33—C32—C31	120.44 (12)
C2—N2—C12	116.39 (10)	C33—C32—H32	119.8
C3—N3—C25	116.16 (10)	C31—C32—H32	119.8
C4—N4—C30	116.49 (10)	C34—C33—C32	120.24 (12)
C5—N5—C43	116.77 (10)	C34—C33—H33	119.9
C6—N6—C48	116.57 (10)	C32—C33—H33	119.9
N1—C1—C2	121.85 (10)	C33—C34—C35	119.71 (12)
N1—C1—C6	118.82 (10)	C33—C34—H34	120.1
C2—C1—C6	119.30 (10)	C35—C34—H34	120.1
N2—C2—C1	122.22 (10)	C34—C35—C36	120.55 (12)
N2—C2—C3	117.20 (10)	C34—C35—H35	119.7
C1—C2—C3	120.56 (10)	C36—C35—H35	119.7
N3—C3—C4	122.47 (10)	C35—C36—C31	120.13 (12)
N3—C3—C2	117.23 (10)	C35—C36—H36	119.9
C4—C3—C2	120.26 (10)	C31—C36—H36	119.9
N4—C4—C3	121.81 (10)	C42—C37—C38	118.35 (11)
N4—C4—C5	118.87 (10)	C42—C37—C28	120.41 (11)
C3—C4—C5	119.32 (10)	C38—C37—C28	121.10 (11)
N5—C5—C6	121.65 (10)	C39—C38—C37	120.58 (13)
N5—C5—C4	117.87 (10)	C39—C38—H38	119.7
C6—C5—C4	120.48 (10)	C37—C38—H38	119.7
N6—C6—C5	121.95 (10)	C40—C39—C38	120.43 (13)
N6—C6—C1	118.34 (10)	C40—C39—H39	119.8
C5—C6—C1	119.70 (10)	C38—C39—H39	119.8
N1—C7—C8	120.43 (11)	C39—C40—C41	119.76 (12)
N1—C7—C12	121.41 (10)	C39—C40—H40	120.1
C8—C7—C12	118.14 (10)	C41—C40—H40	120.1
C9—C8—C7	122.09 (11)	C40—C41—C42	119.84 (13)
C9—C8—H8	119.0	C40—C41—H41	120.1
C7—C8—H8	119.0	C42—C41—H41	120.1
C8—C9—C10	119.46 (10)	C41—C42—C37	121.05 (12)
C8—C9—C13	117.04 (11)	C41—C42—H42	119.5
C10—C9—C13	123.45 (10)	C37—C42—H42	119.5
C11—C10—C9	118.05 (10)	N5—C43—C44	119.26 (10)
C11—C10—C19	116.27 (11)	N5—C43—C48	121.46 (11)
C9—C10—C19	125.65 (10)	C44—C43—C48	119.27 (11)
C10—C11—C12	122.52 (11)	C45—C44—C43	121.32 (11)
C10—C11—H11	118.7	C45—C44—H44	119.3
C12—C11—H11	118.7	C43—C44—H44	119.3
N2—C12—C11	118.80 (11)	C44—C45—C46	119.37 (11)
N2—C12—C7	121.68 (11)	C44—C45—C49	118.20 (11)
C11—C12—C7	119.51 (11)	C46—C45—C49	122.40 (10)
C18—C13—C14	118.81 (11)	C47—C46—C45	119.76 (11)
C18—C13—C9	121.17 (11)	C47—C46—C55	119.40 (11)
C14—C13—C9	119.97 (11)	C45—C46—C55	120.83 (10)
C15—C14—C13	120.58 (12)	C46—C47—C48	121.10 (11)
C15—C14—H14	119.7	C46—C47—H47	119.4
C13—C14—H14	119.7	C48—C47—H47	119.4
C16—C15—C14	119.99 (13)	N6—C48—C47	119.90 (11)
C16—C15—H15	120.0	N6—C48—C43	121.16 (11)
C14—C15—H15	120.0	C47—C48—C43	118.94 (11)
C15—C16—C17	119.89 (12)	C54—C49—C50	118.52 (11)
C15—C16—H16	120.1	C54—C49—C45	121.15 (11)
C17—C16—H16	120.1	C50—C49—C45	120.21 (11)
C16—C17—C18	120.19 (13)	C51—C50—C49	120.42 (12)
C16—C17—H17	119.9	C51—C50—H50	119.8
C18—C17—H17	119.9	C49—C50—H50	119.8
C17—C18—C13	120.53 (13)	C52—C51—C50	120.52 (12)
C17—C18—H18	119.7	C52—C51—H51	119.7
C13—C18—H18	119.7	C50—C51—H51	119.7
C20—C19—C24	117.96 (11)	C51—C52—C53	119.49 (12)
C20—C19—C10	122.87 (11)	C51—C52—H52	120.3
C24—C19—C10	118.98 (11)	C53—C52—H52	120.3
C21—C20—C19	120.42 (12)	C54—C53—C52	120.07 (12)
C21—C20—H20	119.8	C54—C53—H53	120.0
C19—C20—H20	119.8	C52—C53—H53	120.0
C22—C21—C20	120.84 (12)	C53—C54—C49	120.96 (11)
C22—C21—H21	119.6	C53—C54—H54	119.5
C20—C21—H21	119.6	C49—C54—H54	119.5
C21—C22—C23	119.57 (12)	C60—C55—C56	118.87 (11)
C21—C22—H22	120.2	C60—C55—C46	120.40 (11)
C23—C22—H22	120.2	C56—C55—C46	120.73 (11)
C22—C23—C24	119.89 (12)	C57—C56—C55	120.45 (12)
C22—C23—H23	120.1	C57—C56—H56	119.8
C24—C23—H23	120.1	C55—C56—H56	119.8
C23—C24—C19	121.27 (12)	C56—C57—C58	120.36 (13)
C23—C24—H24	119.4	C56—C57—H57	119.8
C19—C24—H24	119.4	C58—C57—H57	119.8
N3—C25—C26	118.94 (11)	C59—C58—C57	119.58 (12)
N3—C25—C30	121.74 (11)	C59—C58—H58	120.2
C26—C25—C30	119.32 (11)	C57—C58—H58	120.2
C27—C26—C25	121.72 (11)	C58—C59—C60	120.47 (12)
C27—C26—H26	119.1	C58—C59—H59	119.8
C25—C26—H26	119.1	C60—C59—H59	119.8
C26—C27—C28	119.20 (11)	C55—C60—C59	120.26 (12)
C26—C27—C31	117.00 (11)	C55—C60—H60	119.9
C28—C27—C31	123.74 (11)	C59—C60—H60	119.9
C29—C28—C27	119.15 (11)	Cl1—C61—Cl2	111.51 (7)
C29—C28—C37	118.19 (11)	Cl1—C61—H61A	109.3
C27—C28—C37	122.53 (11)	Cl2—C61—H61A	109.3
C28—C29—C30	121.82 (11)	Cl1—C61—H61B	109.3
C28—C29—H29	119.1	Cl2—C61—H61B	109.3
C30—C29—H29	119.1	H61A—C61—H61B	108.0
N4—C30—C29	120.21 (11)	Cl3—C62—Cl4	111.38 (7)
N4—C30—C25	121.24 (10)	Cl3—C62—H62A	109.4
C29—C30—C25	118.52 (11)	Cl4—C62—H62A	109.4
C36—C31—C32	118.92 (11)	Cl3—C62—H62B	109.4
C36—C31—C27	121.40 (11)	Cl4—C62—H62B	109.4
C32—C31—C27	119.51 (11)	H62A—C62—H62B	108.0
			
C7—N1—C1—C2	0.39 (16)	C25—C26—C27—C28	1.87 (18)
C7—N1—C1—C6	178.51 (10)	C25—C26—C27—C31	−175.41 (11)
C12—N2—C2—C1	3.53 (16)	C26—C27—C28—C29	−4.81 (18)
C12—N2—C2—C3	−174.71 (10)	C31—C27—C28—C29	172.28 (11)
N1—C1—C2—N2	−3.64 (17)	C26—C27—C28—C37	171.17 (11)
C6—C1—C2—N2	178.24 (10)	C31—C27—C28—C37	−11.75 (18)
N1—C1—C2—C3	174.54 (10)	C27—C28—C29—C30	2.96 (18)
C6—C1—C2—C3	−3.57 (16)	C37—C28—C29—C30	−173.19 (11)
C25—N3—C3—C4	1.04 (16)	C4—N4—C30—C29	−177.43 (11)
C25—N3—C3—C2	178.61 (10)	C4—N4—C30—C25	0.63 (16)
N2—C2—C3—N3	1.24 (16)	C28—C29—C30—N4	179.89 (11)
C1—C2—C3—N3	−177.04 (10)	C28—C29—C30—C25	1.78 (18)
N2—C2—C3—C4	178.87 (10)	N3—C25—C30—N4	−2.53 (18)
C1—C2—C3—C4	0.59 (16)	C26—C25—C30—N4	177.19 (11)
C30—N4—C4—C3	1.96 (16)	N3—C25—C30—C29	175.56 (11)
C30—N4—C4—C5	−177.66 (10)	C26—C25—C30—C29	−4.72 (17)
N3—C3—C4—N4	−2.97 (18)	C26—C27—C31—C36	130.02 (13)
C2—C3—C4—N4	179.52 (10)	C28—C27—C31—C36	−47.12 (18)
N3—C3—C4—C5	176.64 (10)	C26—C27—C31—C32	−45.20 (16)
C2—C3—C4—C5	−0.86 (16)	C28—C27—C31—C32	137.66 (13)
C43—N5—C5—C6	3.42 (16)	C36—C31—C32—C33	−1.07 (18)
C43—N5—C5—C4	−177.02 (10)	C27—C31—C32—C33	174.26 (11)
N4—C4—C5—N5	4.35 (16)	C31—C32—C33—C34	0.42 (19)
C3—C4—C5—N5	−175.28 (10)	C32—C33—C34—C35	0.43 (19)
N4—C4—C5—C6	−176.09 (10)	C33—C34—C35—C36	−0.6 (2)
C3—C4—C5—C6	4.28 (16)	C34—C35—C36—C31	−0.1 (2)
C48—N6—C6—C5	2.78 (16)	C32—C31—C36—C35	0.90 (19)
C48—N6—C6—C1	−176.00 (10)	C27—C31—C36—C35	−174.34 (12)
N5—C5—C6—N6	−6.51 (18)	C29—C28—C37—C42	−43.08 (17)
C4—C5—C6—N6	173.94 (10)	C27—C28—C37—C42	140.91 (13)
N5—C5—C6—C1	172.25 (10)	C29—C28—C37—C38	132.67 (13)
C4—C5—C6—C1	−7.29 (16)	C27—C28—C37—C38	−43.34 (18)
N1—C1—C6—N6	7.57 (16)	C42—C37—C38—C39	0.43 (19)
C2—C1—C6—N6	−174.26 (10)	C28—C37—C38—C39	−175.40 (12)
N1—C1—C6—C5	−171.24 (10)	C37—C38—C39—C40	−0.1 (2)
C2—C1—C6—C5	6.93 (16)	C38—C39—C40—C41	−0.2 (2)
C1—N1—C7—C8	−175.81 (10)	C39—C40—C41—C42	0.2 (2)
C1—N1—C7—C12	2.53 (16)	C40—C41—C42—C37	0.2 (2)
N1—C7—C8—C9	177.93 (11)	C38—C37—C42—C41	−0.48 (19)
C12—C7—C8—C9	−0.46 (17)	C28—C37—C42—C41	175.38 (12)
C7—C8—C9—C10	3.64 (18)	C5—N5—C43—C44	−179.04 (11)
C7—C8—C9—C13	−173.85 (11)	C5—N5—C43—C48	2.65 (16)
C8—C9—C10—C11	−2.59 (17)	N5—C43—C44—C45	−174.46 (11)
C13—C9—C10—C11	174.73 (11)	C48—C43—C44—C45	3.89 (18)
C8—C9—C10—C19	175.49 (11)	C43—C44—C45—C46	0.48 (18)
C13—C9—C10—C19	−7.18 (18)	C43—C44—C45—C49	−177.63 (11)
C9—C10—C11—C12	−1.63 (18)	C44—C45—C46—C47	−4.07 (17)
C19—C10—C11—C12	−179.89 (11)	C49—C45—C46—C47	173.96 (11)
C2—N2—C12—C11	178.93 (11)	C44—C45—C46—C55	174.32 (11)
C2—N2—C12—C7	−0.57 (17)	C49—C45—C46—C55	−7.66 (17)
C10—C11—C12—N2	−174.68 (11)	C45—C46—C47—C48	3.20 (18)
C10—C11—C12—C7	4.83 (18)	C55—C46—C47—C48	−175.21 (11)
N1—C7—C12—N2	−2.59 (18)	C6—N6—C48—C47	−176.64 (11)
C8—C7—C12—N2	175.78 (11)	C6—N6—C48—C43	3.29 (17)
N1—C7—C12—C11	177.91 (11)	C46—C47—C48—N6	−178.90 (11)
C8—C7—C12—C11	−3.71 (17)	C46—C47—C48—C43	1.18 (18)
C8—C9—C13—C18	131.14 (13)	N5—C43—C48—N6	−6.33 (18)
C10—C9—C13—C18	−46.24 (17)	C44—C43—C48—N6	175.36 (11)
C8—C9—C13—C14	−46.20 (16)	N5—C43—C48—C47	173.60 (11)
C10—C9—C13—C14	136.41 (12)	C44—C43—C48—C47	−4.71 (17)
C18—C13—C14—C15	1.27 (19)	C44—C45—C49—C54	128.73 (13)
C9—C13—C14—C15	178.68 (12)	C46—C45—C49—C54	−49.32 (17)
C13—C14—C15—C16	−0.6 (2)	C44—C45—C49—C50	−47.16 (17)
C14—C15—C16—C17	−0.3 (2)	C46—C45—C49—C50	134.79 (13)
C15—C16—C17—C18	0.7 (2)	C54—C49—C50—C51	−0.62 (19)
C16—C17—C18—C13	0.0 (2)	C45—C49—C50—C51	175.38 (12)
C14—C13—C18—C17	−0.95 (19)	C49—C50—C51—C52	−0.7 (2)
C9—C13—C18—C17	−178.32 (12)	C50—C51—C52—C53	1.3 (2)
C11—C10—C19—C20	143.00 (12)	C51—C52—C53—C54	−0.6 (2)
C9—C10—C19—C20	−35.12 (18)	C52—C53—C54—C49	−0.8 (2)
C11—C10—C19—C24	−31.82 (16)	C50—C49—C54—C53	1.35 (19)
C9—C10—C19—C24	150.07 (12)	C45—C49—C54—C53	−174.61 (12)
C24—C19—C20—C21	0.87 (18)	C47—C46—C55—C60	−54.54 (16)
C10—C19—C20—C21	−174.00 (11)	C45—C46—C55—C60	127.07 (13)
C19—C20—C21—C22	1.38 (19)	C47—C46—C55—C56	125.28 (14)
C20—C21—C22—C23	−2.2 (2)	C45—C46—C55—C56	−53.11 (17)
C21—C22—C23—C24	0.8 (2)	C60—C55—C56—C57	−0.1 (2)
C22—C23—C24—C19	1.5 (2)	C46—C55—C56—C57	−179.94 (13)
C20—C19—C24—C23	−2.31 (19)	C55—C56—C57—C58	−1.0 (2)
C10—C19—C24—C23	172.76 (11)	C56—C57—C58—C59	0.8 (2)
C3—N3—C25—C26	−178.15 (11)	C57—C58—C59—C60	0.4 (2)
C3—N3—C25—C30	1.57 (17)	C56—C55—C60—C59	1.33 (19)
N3—C25—C26—C27	−177.36 (11)	C46—C55—C60—C59	−178.84 (12)
C30—C25—C26—C27	2.91 (18)	C58—C59—C60—C55	−1.5 (2)

### 2,3,8,9,14,15-Hexaphenyl-5,6,11,12,17,18-hexazatrinaphthylene dichloromethane disolvate (2) Hydrogen-bond geometry (Å, º)

**Table:**

*D*—H···*A*	*D*—H	H···*A*	*D*···*A*	*D*—H···*A*
C61—H61*A*···N1^i^	0.99	2.46	3.2380 (17)	135
C61—H61*B*···N2	0.99	2.40	3.2745 (17)	146
C61—H61*B*···N3	0.99	2.61	3.4923 (18)	149
C62—H62*A*···N4	0.99	2.58	3.2547 (17)	126
C62—H62*A*···N5	0.99	2.46	3.4381 (17)	169

Symmetry code: (i) −*x*, −*y*+1, −*z*+1.
